# Optical Technologies for the Improvement of Skin Cancer Diagnosis: A Review

**DOI:** 10.3390/s21010252

**Published:** 2021-01-02

**Authors:** Laura Rey-Barroso, Sara Peña-Gutiérrez, Carlos Yáñez, Francisco J. Burgos-Fernández, Meritxell Vilaseca, Santiago Royo

**Affiliations:** Centre for Sensors, Instruments and Systems Development, Universitat Politècnica de Catalunya, 08222 Terrassa, Spain; laura.rey.barroso@upc.edu (L.R.-B.); sara.pena.gutierrez@upc.edu (S.P.-G.); carlos.rene.yanez@upc.edu (C.Y.); francisco.javier.burgos@upc.edu (F.J.B.-F.); meritxell.vilaseca@upc.edu (M.V.)

**Keywords:** skin cancer, melanoma, multispectral imaging, 3D topography, optical feed-back interferometry, confocal microscopy, optical coherence tomography, polarimetry, machine learning

## Abstract

The worldwide incidence of skin cancer has risen rapidly in the last decades, becoming one in three cancers nowadays. Currently, a person has a 4% chance of developing melanoma, the most aggressive form of skin cancer, which causes the greatest number of deaths. In the context of increasing incidence and mortality, skin cancer bears a heavy health and economic burden. Nevertheless, the 5-year survival rate for people with skin cancer significantly improves if the disease is detected and treated early. Accordingly, large research efforts have been devoted to achieve early detection and better understanding of the disease, with the aim of reversing the progressive trend of rising incidence and mortality, especially regarding melanoma. This paper reviews a variety of the optical modalities that have been used in the last years in order to improve non-invasive diagnosis of skin cancer, including confocal microscopy, multispectral imaging, three-dimensional topography, optical coherence tomography, polarimetry, self-mixing interferometry, and machine learning algorithms. The basics of each of these technologies together with the most relevant achievements obtained are described, as well as some of the obstacles still to be resolved and milestones to be met.

## 1. Introduction

Skin cancer is the uncontrolled growth of abnormal skin cells that occurs when unrepaired deoxyribonucleic acid (DNA) genetic defects lead them to multiply and form malignant tumors [1]. The great majority of skin cancer etiologies usually are classified into two large groups: melanoma and non-melanoma skin cancers. Melanoma represents 4% of skin cancer lesions and is the most aggressive and lethal of all forms [2]. It originates from the melanocytes, the pigment-making cells of the skin, and it is more likely to grow and spread if left undertreated; hence, its early diagnosis and treatment is crucial for dermatologists. For this reason, work-related authors have given priority to the detection of melanoma. Apart from the carcinogenic form, melanocytes can also form benign growths called moles [3]. On the other hand, the non-melanoma group includes Basal Cell Carcinoma (BCC), which is the most common type (about eight out of 10 of the skin cancers), and Squamous Cell Carcinoma (SCC), which is the second most common form (about two out of 10). These cancers are most often found in body areas exposed to the sun, such as the head, neck, and arms, but they may appear elsewhere. BCCs tend to grow slowly and it is unusual that they spread to other parts of the body; however, if left untreated, they can invade the surrounding areas. Regarding SCCs, they are more likely to grow into deeper layers of skin and spread to other parts of the body, although this is uncommon [3].

The incidence of both melanoma and non-melanoma skin cancers has increased over the past decades, being one in three cancers diagnosed as skin cancer [4]. Currently, between 2 and 3 million non-melanoma skin cancers and 132,000 melanoma skin cancers occur globally each year. The World Health Organization estimates that 60,000 people die every year because of long sun exposures: 48,000 from melanoma and 12,000 from other skin cancer types. The main factors that predispose to the development of skin cancer, especially melanoma, seem to be connected with recreational exposure to the sun and a history of sunburn [4].

Nowadays, visual inspections by the naked eye and dermoscopy still comprise the first response gold standard to diagnose the disease, but they fail in the correct discrimination of lesions in a relevant percentage of the cases. These inspections base the diagnosis in the so-called *ABCDE* rule, which outlines warning signs of the most common types of melanoma: *A* is for asymmetry, *B* is for border irregularity, *C* is for color, *D* is for the diameter, and *E* is for its evolution [5]. A dermoscope is a handheld device that includes a magnifying lens and a white, uniform illumination with polarized light. However, this technique produces a large number of false positives; that is, benign lesions erroneously classified as malignant. Thus, the real gold standard is histological examination, which requires the surgical excision of the tumor (biopsy), thus contributing to the high direct annual costs for the diagnosis and treatment of skin cancer [6]. In this surgical procedure, specialists need to apply anesthesia, make the incision, and control the surgery. Afterwards, a specialist in histology treats the sample and identifies what is abnormal under the microscope. Apart from the cost, the multiple physicians involved make it a long-term process, taking two or three weeks from the extraction of the lesion until the final diagnosis is in place [7].

Due to the large number of affected people, many authors have put their efforts in detecting skin cancer quantitatively and non-invasively through optical and photonic devices. This is reinforced by the fact that skin is the external layer of the body, where optical techniques have easy access. In particular, they can be useful to face the challenge of early detection of skin cancer and to improve the current diagnostic tools such as dermoscopy, thus avoiding unnecessary biopsies. The use of these techniques is actually increasing because they can offer both spatial and spectral data, among other relevant information of tissue. 

The imaging modalities commonly used are diverse, but among the most popular techniques there are confocal microcopy, Multispectral (MS) imaging, 3D topography, Optical Coherence Tomography (OCT), Self-Mixing Interferometry (SMI) and Polarized Imaging (PI). In parallel to the development of these modalities, the use of machine learning algorithms for the automated discrimination of skin lesions has become a popular resource among authors, by means of collecting extensive datasets of diverse nature to feed those algorithms. This work is intended to be an extensive review on the many optical techniques, as well as learning algorithms that are applied to the analysis of skin in dermatology, particularly for the detection of skin cancer.

## 2. Confocal Microscopy

**Figure 1:**
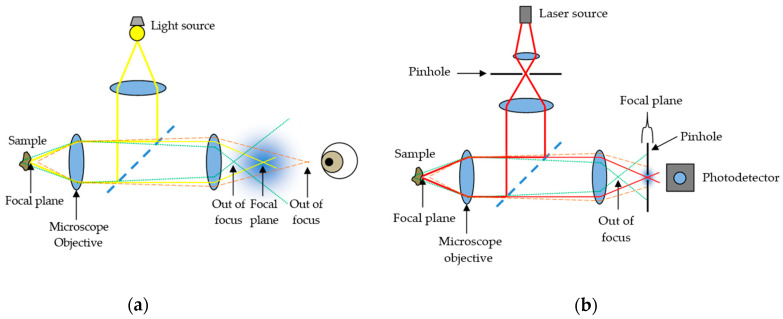
Comparison between (**a**) conventional optical microscopy and (**b**) confocal laser scanning microscopy.

Confocal microscopy, most frequently Confocal Laser Scanning Microscopy (CLSM) or Laser Confocal Scanning Microscopy (LCSM), is an optical imaging technique for increasing the optical resolution and contrast of a micrograph by means of using a spatial pinhole to block out-of-focus light or glare in image formation. Confocal microscopy offers several advantages over conventional wide field optical microscopy, including the ability to control depth of field, elimination or reduction of background information away from the focal plane (that leads to image degradation), and the capability to collect serial optical sections from thick specimens whose thickness exceeds the immediate plane of focus (Figure 1).

In modern confocal systems, stacks of optical sections can be recorded over time, enabling the observation of time-dependent processes within 3D volumes (4D microscopy). In skin research, confocal techniques are commonly used for the in vivo investigation of surfaces, tissue structure, or cell cultures as well as for ex vivo histological studies. Among researchers, CLSM is becoming increasingly popular due to the relative ease with which extremely high-quality images can be obtained, where it is possible to observe the cellular size and shape, the epidermal structure, the dermal collagen morphology, and the keratin [8], serving as a tool for the analysis of equivocal skin lesions.

The spatial resolution of the system, which is the system’s ability to resolve two adjacent point objects in the lateral and axial directions, depends on the size of the illumination spot. The size of the illumination spot on the sample ranges from approximately 0.25 to 0.8 micrometers in diameter, which accounts for the lateral resolution (depending upon the objective numerical aperture), and 0.5 to 1.5 micrometers in depth at the brightest intensity, accounting for axial resolution [9]. The maximum depth within the sample from which optical sections with acceptable signal-to-noise ratio can be recorded depends on the imaging wavelength (excitation and emission), the working distance of the objective, and the optical density of the sample. In general, it is considered to be about 70 μm. 

Regarding confocal microscope architectures, there are two outstanding families: slow scan, single beam confocal microscopes and faster Nipkow systems using a multiple beam approach. Single beam instruments scan the sample point by point in a raster pattern, working with galvanometer-driven mirrors. These instruments work with different laser lines for excitation: Argon ion lasers (488 and 514 nm), Argon–Krypton lasers (476, 488, 576, and 623 nm) and Helium-Neon lasers (543 and 632 nm). They use Photomultiplier Tubes (PMT) or hybrid detectors, ideally using one separate detection channel for each dye. On the other hand, Nipkow systems are equipped with a spinning disk behind the light source, containing an array of micro lenses or holes, which divide light into multiple beams simultaneously focused at the sample. These multiple beam instruments can work with normal arc-discharge lamps, and the signal can be captured in video rate with an ordinary charge-coupled device (CCD) camera. The former is better to image thick and dense tissue (no cross talk between multiple beams), and the latter is best for the investigation of rapid processes in living cells or tissue.

Looking at the number of different imaging modes in confocal microscopy, two basic operating modes can be differentiated: reflectance and fluorescence CLSM. They both have been used to conduct different research objectives in the field of dermatology.

### 2.1. Reflectance CLSM 

Reflectance CLSM imaging uses a detection wavelength identical to the excitation wavelength. Here, light from reflection and scattering events is recorded, but no fluorescence signal at wavelengths longer than the excitation one is used. In this imaging mode, the image contrast derives mainly from refractive index changes, and the highest signal intensity usually originates from the transition between air and the sample surface. Thus, this mode is ideal for visualizing the topography of surfaces. Moreover, by using an immersion medium different from air (immersion oil, glycerin, or water), also structures lying under the sample surface can be observed. Since the reflectance configuration does not require staining of the sample, this mode can easily be used for in vivo imaging of skin surfaces and pathology, too.

### 2.2. Fluorescence CLSM 

Fluorescence CLSM imaging uses a detection wavelength longer than the excitation one, since fluorescence emission displays a red shift characteristic for the respective fluorophore. This fluorescence is emitted either by skin autofluorophores, e.g., lipofuscin or NAD(P)H, or by fluorescent dyes that have been introduced into the sample following a specific staining protocol. The specificity of these staining techniques ranges from the general depiction of skin structure up to the highly specific staining of single target molecules in skin cells. Using a combination of fluorescent dyes with different emission characteristics, also different target structures can be simultaneously stained and visualized in parallel. In standard fluorescence microscopy, such investigations are mostly restricted to skin sections with a maximum thickness of ≈10 μm, since the superposition of stained structures in thicker samples impedes any discrimination of discrete labels.

The former modality, reflectance CLSM, which is applied in vivo, has become an important adjunct to clinical examination, dermoscopy, and histopathology assessment in the diagnosis and management of melanoma and other skin cancers [10]. For instance, it has been applied at the patient’s bedside for lateral margin detection because of its capability of exploring the skin at the cellular level, enabling the identification of tumor characteristics [11]. Additionally, the non-invasive nature of CLSM allows evaluations of skin lesions over time, thus enabling the investigation of dynamic physiological or pathological processes in vivo and at different time points [12]. Nevertheless, both modalities (especially, fluorescence CLSM) are applied ex vivo to tissue biopsies, and there is in-depth research on how to best preserve the properties of living tissue for as long as possible when preparing samples for visualization. For the 3D visualization of skin samples at high resolution, it is essential to preserve the skin structure in a near-to-life state. In the latter modality, fluorescence CLSM, the use of fluorescent dyes for skin staining even allows the 3D investigation of the structure in skin biopsies due to the possibility of optical sectioning in confocal systems. Hence, the CLSM investigation of samples stained with fluorescent labels enables the precise localization of cells or their constituents within an intact 3D environment [13]. In fact, ex vivo CLSM has also been used on freshly excised tumors in the operating room with different fluorophores allowing for tumor demarcation [11]. Some examples of contributions of CSLM imaging to the diagnosis of skin cancer are presented below, and in addition, summarized in Table 1.

### 2.3. Contributions of CLSM Imaging

By means of reflectance CLSM, Pellacani et al. [14] have attempted to describe and characterize the cytological and architectural aspects of cell clusters in melanocytic lesions and to correlate them with routine histopathology.

In addition, Guitera et al. have conducted extensive research on the possibilities of reflectance CLSM evaluating melanomas [15] and basal cell carcinomas [16] by the implementation of a two-step scoring methodology. In the first study, they managed to improve the specificity compared with dermoscopy and, in addition, they were able to classify 100% of the non-biopsied control nevi population as benign. In the second study, they defined a multivariate algorithm to improve the diagnostic accuracy of basal cell carcinomas with very high sensitivity and specificity values. Under the microscope, they identified characteristic features of BCCs, such as telangiectasia and convoluted vessels, basaloid nodules, and epidermal shadowing corresponding to horizontal clefting.

Segura et al. [17] analyzed features of melanocytic and non-melanocytic skin tumors by reflectance CLSM and performed univariate and multivariate analyses to determine the association of Reflectance Confocal Microscopy (RCM) features with tumor types. 

In addition, there are several studies on the assessment of Actinic Keratosis (AK), which is a precancerous condition that is very useful to detect in order to prevent skin cancer. Ulrich et al. [18] applied in vivo reflectance CLSM for the evaluation of AKs in correlation with routine histology. RCM features of AK included parakeratosis, architectural disarray, and keratinocyte pleomorphism. The presence of architectural disarray and cellular pleomorphism appeared to be the best predictor of AK. Horn et al. [19] also analyzed AKs under reflectance CLSM; the confocal images were rated by four independent derma oncologists, who were able to identify the distinct morphologic features from AKs. The overall sensitivity and specificity achieved were very high, showing the potential of reflectance CLSM as a non-invasive monitoring tool.

Ex vivo fluorescence CLSM is used more and more on a routine basis in the clinic. Freshly excised skin tumors are analyzed under fluorescence CLSM in the operating room. Different fluorophores, such as fluorescein, Nile blue, patent blue, methylene blue, and acridine orange, can be used at different wavelengths. However, acridine orange is the most commonly used because of its capability to provide an excellent contrast [11]. Most studies are performed on the commercial system VivaScope 2500^®^ (MAVIG GmbH, Munich, Germany). The laser illumination wavelength is 488 nm. The depth is manually adjusted to image the surface. It provides optical sectioning of approximately 1.5 μm and resolution of approximately 0.4 μm. In addition, most studies have been performed in carcinomas, mostly in BCCs.

Gareau et al. have obtained confocal mosaics in excised skin lesions [20] and in particular of BCCs [21] to minimize the need for frozen histology. They used acetic acid as a reflectance contrast agent to enhance nuclear-to-dermis contrast. They compared the mosaics to histology, which requires 9 min instead of 20–45 min per excision for preparing frozen histology, and thus may provide a means for rapid pathology-at-the-bedside to expedite and guide surgery.

Abeytunge et al. [22] is another group of authors that have used fluorescence CLSM attempting to rapidly image larger areas of tissue. The limited field of view of high-resolution microscopes requires the merging of multiple images that are taken sequentially to cover a large area. This merging or mosaicing of images requires long acquisition and processing times, and it produces artifacts. They developed a methodology to reduce both time and artifacts, to image large areas of excised tissue with sub-cellular detail.

## 3. Multispectral Imaging

Multispectral (MS) imaging is another imaging modality widely used by authors in the field of dermatology. MS imaging systems allow the reflected or transmitted light from skin to be measured through several spectral bands of the electromagnetic spectrum range with high spatial resolution. Spectral properties of the skin are mainly caused by groups of organic molecules called chromophores, such as melanin, hemoglobin, water, beta-carotene, collagen and bilirubin; and they are known to differ among skin lesions of different etiologies. The reflected and transmitted light that MS systems can capture contains information on these chromophores. MS imaging systems can sequentially illuminate the skin with light at different spectral bands and collect the reflected light at the sensor, or they can sequentially filter the reflected or transmitted light coming from the skin before reaching the camera sensor. These two approaches (active and passive) for collecting light give systems certain advantages and disadvantages for their application into skin analysis: active systems illumination are usually based on Light Emitting Diodes (LEDs), which are low cost, have a small size, long durability, and low energy consumption. They allow optical designs more compact than passive variants. Nevertheless, passive systems provide continuous spectral sampling as they are based on electro-optical devices, such as liquid crystal (LCTF) or acousto-optic (AOTF) tunable filters.

The final outcome of these MS systems is known as a spectral cube, which contains all the spatial and spectral data from the sample under analysis. Depending on how the spectral cube is built, MS imaging devices are classified into different categories [23]. Point or line scanning systems, in which the spectrum of a single point or a single line is captured sequentially, require the scanning of the sample in one or two spatial directions. Unfortunately, these two methods are not suitable for the in vivo analysis of the skin. Spatial scanning MS imaging systems are very time-consuming and require a complex mechanical configuration to scan the whole area of the sample [24]. They would be suited for measuring skin grafts, or failing that, skin replicas. Nonetheless, many of the in vivo absorption and reflectance properties that the skin has would be corrupted, and on the other hand, reproducing the same qualities on a skin replica is still too complex. Skin has a complex structure and is composed of several organic elements, such as blood, fat, or melanin pigment, among others; and the relative composition varies strongly among individuals.

This is why skin is always analyzed in vivo by means of spectral imaging, and therefore, all MS systems designed for skin analysis are of the type of area scanning or snapshot imaging systems.

### 3.1. Area Scanning MS Systems

Area scanning systems or staring imagers capture a two-dimensional data matrix (full resolution image) of the skin at one spectral channel at a time. A complete spectral cube is obtained by collecting a sequence of these images. 

### 3.2. Snapshot MS Systems

Snapshot systems provide an even faster approach, intended to record both spatial and spectral information with only one exposure. Nevertheless, since the sensor area holds all the information from the scene, spatial or spectral resolution is very limited. 

The most used digital sensors in spectral imaging systems, especially in area scanning ones, are the CCD and CMOS cameras. CMOS sensors are currently preferred due to their simpler manufacturing. They have a spectral response in the visible (VIS) (400–700 nm) up to the near-infrared (NIR) (800–1000 nm) [25]. However, multispectral, extended near-infrared (exNIR) optical imaging is nowadays available thanks to new Indium Gallium arsenide (InGaAs) cameras with high quantum efficiency within 900–1600 nm [26].The most commonly selected spectral bands for skin analysis based on the absorption curves of the principal chromophores are the 410−415 nm band, for melanin and oxygenated/deoxygenated hemoglobin maximum absorption, where melanin absorbs much of the blue light, and thus, it is perceived as brown, whereas hemoglobin absorbs mostly blue and green wavelengths with peaks at 400 and 550 nm, and therefore, it has a reddish appearance; the 440−460 nm band, where bilirubin has its maximum absorption; the 460−485 nm band of deoxygenated hemoglobin minimum absorption; the 650−670 nm band of oxygenated hemoglobin minimum absorption, and additional bands in the NIR to obtain information about the deeper content in the skin [27,28,29,30,31,32,33,34,35,36]. The choice of bands beyond 800 nm into the NIR and exNIR regions enables deeper in vivo optical imaging as photons penetrate deeper into tissue layers where information about damage due to ultraviolet radiation and water content (related to tumor angiogenesis, that is, the growth of blood vessels around the malignant lesion) may be found [37]. This has sparked interest among biologists in this relatively unexplored spectral region. Some examples of contributions of MS imaging to the diagnosis of skin cancer are presented below and summarized in Table 2.

### 3.3. Contributions of MS Imaging

Tomatis et al. [36] made use of a monochromator coupled to a fiber bundle to disperse the reflected light coming from the skin into fifteen spectral bands from 483 to 950 nm. Segmentation, parameter extraction, and data reduction were performed to feed a neural network classifier to perform the classification of the lesions as excision-needing or reassuring [38].

Bekina et al. [30] analyzed lesions under a multispectral system with four different spectral bands, each one to obtain information from specific structures of the skin: 450 nm for superficial layers, 545 nm for blood distribution, 660 nm for melanin detection, and 940 nm for the evaluation of deeper skin layers. They proved that hemoglobin absorption was higher in pathological lesions. In addition, these same authors [39] developed a system with a CCD and a LCTF, with halogen lamps as a system of illumination, where wavelengths of 540, 650, and 950 nm were selected to differentiate between melanoma and nevi.

Jakovels et al. [40] used an LCTF and an imaging camera Nuance (Cambridge Research & Instrumentation, Inc., Hopkinton, MA, USA) based on stabilized halogen lamp ring-light to make MS imaging in the range from 450 to 950 nm. The principal component analysis (PCA) of the spectral parameters resulted in a clear separation for malignant melanomas and pigmented nevi. Kim et al. [41] miniaturized a MS imaging system (range 440 to 690 nm) for its installation on a smartphone camera. It showed a great potential in the quantitative diagnosis and management of skin lesions.

Stamnes et al. [32] made use of 12 fixed LED lamps placed at different angles to obtain MS multi-angle images to enhance the capability to retrieve depth information. The study attempted the discrimination among malignant and benign pigmented lesions.

**Figure 2:**
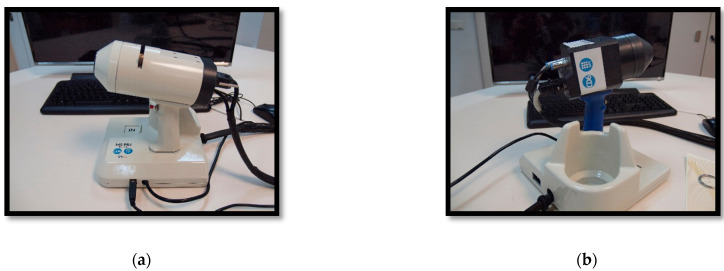

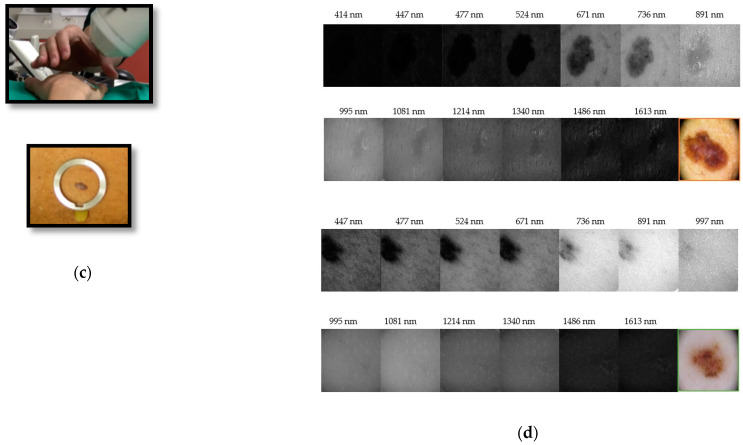
From authors Rey-Barroso et al. [43], multispectral (MS) imaging devices (**a**) General view of the previously developed handheld visible to the near-infrared (VIS-NIR) MS device. (**b**) General view of the handheld extended near-infrared (exNIR) MS device. (**c**) Clinical measurement and metallic ring, which is glued to the patient’s skin for the tip of both systems to place them in the same position and parallel to the skin, without making any contact. (**d**) MS images of a melanoma (top) and a nevi (bottom). The structure of the melanoma is still visible at the longest wavelengths.

Delpueyo et al. [28] (Figure 2) proposed an LEDs-based MS imaging system with eight different wavelengths (414 to 995 nm). Using the spatial distribution of color and spectral features, they allowed an improvement in the detection of skin cancer lesions, specifically melanomas and BCCs.

In addition, other imaging modalities have been combined with MS imaging for the successful discrimination of skin lesion etiologies. 

Vasaturo et al. [42] proposed the combined use of microscopy with MS imaging. They analyzed the presence of tumor-infiltrating lymphocytes (TIL), pathological biomarkers, in human primary melanomas. MS images were loaded into a user trainable software where, per cell and per cellular compartment, the optical intensity values were calculated to score cell positivity for each specific marker.

Focusing on MS, exNIR optical imaging, Rey-Barroso et al. [43] (Figure 2) developed an MS imaging system based on an InGaAs camera and LEDs in the 995 nm to 1613 nm range. Outcomes of this device were combined with those of Delpueyo et al. [28] to provide spectral and spatial information to discriminate between melanomas and nevi.

Additionally, Godoy et al. [26] used a longwave infrared (LWIR) camera to make dynamic thermal imaging (DTI) of skin lesions. The LWIR camera uses a focal-plane array of quantum-well infrared photodetectors. The thermal infrared image sequences acquired allowed the discrimination among benign and malignant skin conditions.

Fioravanti et al. [37] employed a sensor that did not make imaging per se, but recorded the IR spectrum from 3330 to 3570 nm of skin biopsies.

## 4. Three-Dimensional Topography

Three-dimensional (3D) topography imaging techniques are able to retrieve pointwise information on the height of skin surface, hence acquiring height maps of the surface of the lesions from which skin relief can be reproduced. Skin relief, also referred to as surface texture or topography, is an important biophysical feature that can sometimes be difficult to appreciate with the naked eye. 

According to the classification given by Hashimoto [44], the surface pattern of the human skin can be divided into primary, secondary, tertiary, and quaternary structures. The range of the skin roughness value, as expected, is mainly determined by the primary and secondary structures, which consist in deep lines or furrows from 5 to 100 μm in depth; these can be examined by mechanical profilometry with a probe or stylus.

The tertiary and quaternary structures do not visibly contribute to the roughness parameters but cause light to be reflected diffusively. In order to evaluate these fine structures, optical techniques such as laser or focus detection based systems, and triangulation-based systems should be employed [45].

In domains in which the dimensional inspection of surfaces was more frequently and longtime applied, stylus contact systems and interferometric systems were preferred. However, in general, researchers prefer non-invasive techniques for the inspection of skin, such as laser or focus detection based systems, and triangulation-based systems, that are able to retrieve the height of a sample by means of the principle of triangulation. Some studies have made use of skin replicas of silicon rubber where the skin surface is imprinted on, so roughness parameters may be recovered. Replica-based methods are numerous, were the first to be implemented, and are still commonly used despite the recent advances in in vivo topography imaging [46]. Hence, they are still worth-mentioning.

### 4.1. Replica-Based Methods for Skin 3D Imaging

There are mainly two approaches that are used to measure skin replicas: The first, line profiling measurements, are contact methods that use a small probe to detect the peaks and valleys and produce a quantitative height profile. The highest vertical and lateral resolutions achieved with line profiling methods are 0.05 and 0.02 μm, respectively [47].

The second, areal topography methods, are based on optical approaches to create two dimensional topographic images. The ones most used are as follows.

#### 4.1.1. Microtopography

Microtopography is very well-suited to measure the anisotropy of skin furrows [48] and the degree of irregularity in the skin pattern [49]. The skin replica is placed on a reference surface and scanned by moving the sample at equal precise increments under a light beam, projected forming an oblique angle. At each sampled point, the position of the spot is viewed by one or two cameras from a different angle. At each scanning step, the spot shift regarding the start position is calculated, and the thickness or height and the inspected surface points are computed. Large incident angles provide the best resolution but also generate undesired shadows and reflections that interfere with the measurement.

#### 4.1.2. Optical Profilometry

Optical profilometry based on the autofocus principle is the most used optical technique in skin cancer detection. The mechanical probe or stylus aforementioned is substituted by an optical probe. An illumination-detection system is focused on a flat reference plane. Any relief variation will result in image defocusing and decrease of the signal captured by a detector. Then, automatic refocusing is proceeded by shifting the focusing lens in the vertical direction. This shift is measured at each point and then converted to surface height distribution. The precision of laser profilometers is very high, but they require long periods of time to complete the sampling measurements on skin replicas (about 2 h) [50]. In addition, a study showed that the roughness value obtained is sensitive to the spatial frequency cut-off and sampling interval during the signal processing [51].

The highest vertical and lateral resolutions achieved for these optical techniques are 1 nm and 1 μm, respectively [50,51]. 

Within replica-based methods, one also may find area-integrated techniques, which are optical-based methods that capture an area-based signal and relate it directly to one or more statistical parameters without considering point-wise surface information. Despite the fact that replica-based methods are reliable and provide high resolutions, in clinical environments, they are not convenient due to the long scanning times and the distortions obtained during skin relief reproduction. For these reasons, great efforts have been made to improve the performance of 3D in vivo methods, which carry out fast non-contact analysis.

#### 4.2. 3D Systems for In Vivo Skin Analysis

According to the literature, the existing in vivo methods to analyze the skin topography can be divided into three groups.

The first one, videoscopy, is an imaging technique that provides 2D grayscale micro- [52] or macro- [53] photographs for skin texture analysis. The second one, capacitance mapping, is based on capacitive pixel-sensing technology [54] and is an area-integrating approach that images a small area of about 50 μm and exposes skin pores, primary and secondary lines, and wrinkles. 

However, these two first methods do not retrieve height maps, but rather a visual distribution of texture. Both approaches cannot quantify roughness according to the ISO standards [55] and therefore are rarely applied in modern approaches. Laser speckle is another method that does not allow surface profiling but provides a fast, low-cost surface assessment. Speckle is an apparently random distribution of intensity of coherent light that arises by scattering from a rough surface, which carries information on the surface deformations. Speckle contrast is a numerical value that can be easily measured and is well-described theoretically; as it depends only on the properties of the light source, the surface roughness, and the detector. Different surface parameters can be recovered from the measured speckle contrast. Speckle methods are classified as an area-integrating optical technique for direct measurements [46,56,57].

To the best of our knowledge, the only technique widely used today for in vivo skin analysis that quantifies roughness according to ISO standards is the one based in fringe projection topography.

Fringe projection areal topography is based on the triangulation measuring principle combined with light intensity modulation using sinusoidal functions [58]. With the advent of high-resolution CCD cameras and projectors, cost-effective and real-time frame grabbers and powerful image processing software, fringe projection was enhanced by the use of a whole fringe pattern covering the skin sample instead of using a single laser point. Avoiding point-wise scanning, the acquisition time is reduced to a few seconds, which makes this approach ideal for in vivo measurements [59,60]. This technique has also been applied to skin replica micro-relief investigation [47,61,62].

Some examples of contributions of 3D topography to the diagnosis of skin cancer are presented below and also summarized in Table 3.

### 4.3. Contributions of 3D Topography

Gorpas et al. [59] designed a binocular machine vision system for non-melanoma skin cancer 3D reconstruction. Two progressive scan digital color CCD cameras with two telecentric lenses to reduce most distortion factors were used, and a custom structured light projector was included. Illumination from a non-ultraviolet blue light source (dominant wavelength at 440 nm) was delivered to a high-quality printed film through a fiber bundle. They used a 3D reconstruction algorithm that retrieved a very high precision profile, providing for objects of known volume a standard deviation of only 0.0313 mm.

Moore et al. [63] designed a system of structured light projection to measure changes in the 3D topology of the body during irradiation in radiotherapy, which can be applied also to changes in the skin surface. The optical sensor consists of a primary He-Ne laser source and an integrated, pole-mounted optical array made of an interferometer head, adjacent triangulation spot-laser and zoom-lens, and a narrow-band filtered CCD camera. The laser uses radiation-resistant quartz fibers to couple to the interferometer, where the light is split and injected into the twin fiber interferometer. The twin fibers introduce a path length difference to produce and project a divergent pattern of red fringes onto the surface.

**Figure 3:**
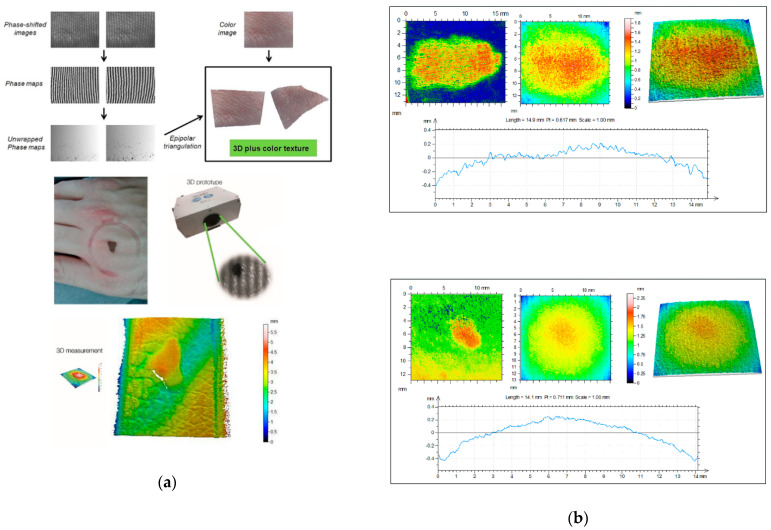
From Rey-Barroso et al. [65], (**a**) 3D fringe projector. The fringe images are reconstructed and unwrapped to obtain 3D images with superimposed color texture. A pigmented lesion and its heights maps are shown below. (**b**) Three-dimensional (3D) topographical images of a melanoma (top) and a nevi (bottom). The melanoma seems to have a rougher profile.

Ares et al. [64] developed a 3D scanner that incorporates two megapixel stereoscopic cameras, which are placed at a certain distance from each other and oriented at a specific angle. In the middle, they placed a picoprojector (Figure 3). It also incorporated a color camera to take a conventional RGB image of the lesions. Rey-Barroso et al. [65] used the same 3D imaging scanner to obtain morphological parameters of skin lesions related to the perimeter, area, and volume with micrometric precision and found significant differences between melanoma and nevus.

Other interesting studies have also been performed on skin replicas, measuring indirectly the 3D profile characteristics of the skin. Korn et al. [66] have evaluated how sun exposure affects skin roughness by measuring negative imprints of the skin surface obtained using a two-component silicone rubber and catalyst system. The replicas were measured by means of a commercial 3D optical profilometer. 

Fringe projection has also some drawbacks, which are essentially due to the volume effects caused by the interference of backscattering from the skin tissue, and it is more sensitive than other modalities to micro-movements of patients. In order to achieve greater accuracy and versatility, several non-contact approaches have been suggested: two-frequency shadow moiré, moving gratings, phase-shifting holographic moiré, slit beam scanning moiré, color gratings, and the use of computer generated gratings [67].

Moore et al. [63] designed a system with similar sensor and a multi-color fringe projection source for avoiding gaps in surface displays due to shadowing and for the construction and quantitative display of wrap-round body features.

In addition, several fringe projection 3D scanners are commercially available. Currently, two devices based on fringe projection are available on the market specifically oriented to skin measurements: PRIMOS^®^ (GFMesstechnik GmbH, Berlin, Germany) and DermaTOP^®^ (Breuckmann, Teltow, Germany). The main difference between them is in how the fringe patterns are produced: PRIMOS^®^ uses micro-mirrors [58], while DermaTOP^®^ uses a template for the shadow projection [61]. Even though performances reported by both systems are similar, DermaTOP^®^ shows the highest performance reaching 2 μm for vertical resolution and 15 μm for lateral resolution with an acquisition time less than 1 second. Some in vivo studies for skin analysis that used these commercial systems can be found in the literature.

## 5. Optical Coherence Tomography

Optical Coherence Tomography (OCT) is another non-invasive imaging technique that can be used for in vivo skin analysis, obtaining real-time, cross-sectional evaluations of the skin. By the use of low-coherence interferometry with an NIR light source, it is possible to detect the light waves backscattered at index of refraction mismatches present in the internal structure of the skin. The backscattered light from a given depth in the tissue is recombined with a static reference signal; and when both path lengths are matched within the so-called coherence length of the light source, an interference occurs, which may be of micrometric size. In this way, it is possible to detect signals from different depths and determine from which depth in the skin the signal comes from. Whereas techniques such as confocal CLSM or MS are limited to imaging the topmost 100–200 micrometers due to a number of issues including multiple scattering, numerical aperture and photon absorption [68], OCT can reliably image as deep as a few millimeters. For instance, this allows identifying the epidermis and the dermo-epidermal junction [69], although at a lower lateral resolution than that of CLSM. 

Both two-dimensional (2D) and three-dimensional (3D) images can be created with the technique. Two-dimensional data (b-scans) are obtained by moving the beam across the skin and acquiring data (retrieving vertical, cross-sectional scans); and 3D data are obtained by translating the beam in two directions over a surface area [70]. En face mode (horizontal), which provides similar images to those of dermoscopy and reflectance CLSM, is also available in most clinical instruments.

Another advantage of OCT over other imaging modalities is that it enables repeated imaging of the same unaltered skin sites to observe dynamic events and long-term changes over time. Therefore, it has been used to aid in the diagnosis of clinical and subclinical lesions, which are sometimes unique to OCT given its larger field of view [71].

There are several different types of OCT. Time-domain OCT (TD-OCT) impressed specialists but was soon left in the shade by Frequency-domain OCT (FD-OCT) [72], which offered shorter acquisition times and higher resolution, becoming soon the standard in dermatological research.

### 5.1. FD-OCT

There are two types of FD-OCT systems based on the same fundamental principle, with two different light sources and detection schemes: Spectral Domain OCT (SD-OCT), which utilizes a broadband, low-coherence light source, and Swept Source OCT (SS-OCT), which uses the coherent and narrowband light of a tunable laser [73,74]. 

Unlike TD-OCT, FD-OCT systems have no moving parts and therefore have high mechanical stability and low phase noise. The availability of linear cameras has enabled the development of SD-OCT systems with varying imaging speeds and sensitivities. SS-OCT systems use a swept laser source and a photodetector. Due to the rapid wavelength tuning, high peak powers at each discrete wavelength can be used to illuminate the skin to provide greater sensitivity with little risk of optical damage. Conventional instruments based on FD-OCT technology have a lateral resolution smaller than 7.5 micrometers and an axial resolution smaller than 5 micrometers, a penetration depth of up to 2 mm, and a field of view (FOV) of 6.0 × 6.0 mm [75]. 

**Figure 4:**
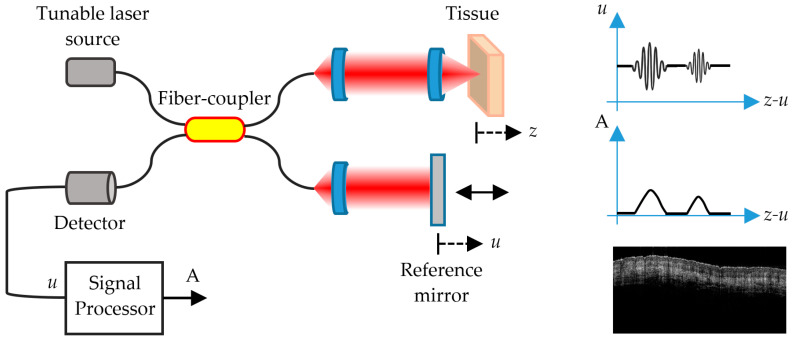
Schematic representation of an SS-OCT imaging system. *u* is the reference signal and *A* is the interference signal that produces the image of the different layers of the skin.

FD-OCT (Figure 4) allows distinguishing skin features that include the epidermis (for which many cancers display an alteration in histology); the dermo-epidermal junction; the upper or papillary dermis; the lower or reticular dermis; the movement of blood vessels through the upper dermis; skin appendages, such as hair follicles and sebaceous glands; and the nail unit and nail plate [72].

A challenge common to any imaging device is the trade-off between high resolution and depth of penetration in the skin. With the trade-off of a more limited penetration depth, High-Definition OCT (HD-OCT) was built on the basis of FD-OCT with the latest technological developments to obtain higher resolution images of the skin, allowing single cells to be visualized. This seems to bridge the gap between conventional FD-OCT imaging and CLSCM. They offer improved axial and lateral resolution of 3 micrometers, with the trade-off of a more limited penetration depth of about 0.57 mm and FOV of 1.8 mm × 1.5 mm [76].

Among OCT modalities, there is a more specific technique known as Dynamic OCT (D-OCT) based on Speckle Variance OCT (SV-OCT), which has recently allowed the visualization of skin microvasculature and the detection of blood flow. The detection of microvasculature growth, known as angiogenesis, is possible by means of D-OCT, hence the name by which this technique is best known is OCT angiography (OCTA). Angiogenesis is a sign of growth and spread of cancers; thus, the visualization of vessel morphology is helpful in improving diagnostic accuracy.

OCTA permits the visualization of vascular patterns in the skin. It involves rapidly repeating OCT scans, and analyzing the statistics of the OCT signal, to detect regions of the OCT images that have changed between these successive scans (such as blood, in vessels) [77,78]. 

The recent studies with the different OCT modalities indicate that the characteristic layering found in normal skin is lost both in non-melanocytic skin lesions (NMSC) and in melanomas (MM). In particular, OCT is considered very helpful for the differentiation of non-pigmented lesions (such as NMSC lesions), since pigmented lesions demonstrate regular scattering patterns that inhibit the differentiation of malignant from benign lesions [72]. The combination of high-resolution and relatively high-imaging depth places OCT in the imaging gap between ultrasound and confocal microscopy [70]. The potential of OCT for tumor margin delineation has to be verified by correlating tumor borders visible in OCT images to the borders identified by invasive techniques, especially in infiltrative types of skin cancer. In addition, OCT has great potential to be used in preoperative treatment planning. Moreover, OCT enables non-invasive treatment monitoring of skin cancers undergoing non-surgical therapies.

In general, the 1300-nm waveband is used in both commercial and experimental OCT systems that cover the water absorption spectral window to obtain satisfactory image depth penetration in skin, at least 500 μm. Other wavelengths have been studied, notably 1050 nm, but all commercial skin imaging OCT devices use the 1300-nm waveband [76].

Other advanced in vivo imaging modalities, such as Raman spectroscopy, have been tested in conjunction with OCT to obtain complementary physiological information about the target tissue. Achieving higher resolution, deeper penetration, or enhanced contrast using multimodal imaging approaches can not only provide additional details but also allow the clinician to immediately assess the 3D morphological and functional characteristics of the target location [79]. Some examples of contributions of OCT to the diagnosis of skin cancer, are presented below and also summarized in Table 4.

### 5.2. Contributions of OCT

Korde et al. [80] evaluated the effect of solar radiation in the skin of patients with minimal and severe solar elastosis. Significant differences were also found between healthy skin and skin with the precancerous condition known as AK. The FD-OCT system used in this study integrated an amplified fiber source with a center wavelength of 1310 nm. A Fourier domain optical delay line was used to achieve an axial scanning rate of 200 per second. The optical signal was detected with a solid-state InGaAs detector with integrated amplifier and bandpass filters. 

Themstrup et al. [81] evaluated the use of VivoSight^®^ (Michelson Diagnostics, Kent, UK) as an OCTA and acquired the in vivo images of the microcirculation in the skin using the build-in OCTA/speckle variance (SV). They found a correlation between the OCTA measurements of skin tumors and other flux measurements.

Weissman et al. [68] described a novel shapelet-based image processing technique for the automatic identification of the upper and lower boundaries of the epidermis. These boundaries are used to measure epidermal thickness and tumor thickness in BCC, which has been studied with OCT and correlated with histology [76]. They used the commercially available Skindex 300 (SkinDex 300, ISIS optronics GmbH, Mannheim, Germany). A detailed review of the newest OCT experimental studies has been performed by Xiong et al. [82].

## 6. Self-Mixing Interferometry

In recent years, Self-Mixing Interferometry (SMI) has been applied to the design of new laser-based biomedical sensors, including sensors for skin analysis and cancer detection in the field of dermatology [83].

SMI, also known as optical feedback interferometry (OFI), is a homodyne detection scheme that is achieved when a very small portion of the beam emitted by a laser source is reinjected back into the laser cavity after interacting with a target. This re-injected field recombines with the resonant modes of the laser, causing a variation in the optical output power and the voltage across the laser terminals, parameters from where the interferometric signal can be acquired indistinctly. The interaction impinges the back-reflected field with information related to the state of target, such as its absolute distance, velocity of displacement, vibration frequency, and complex reflectivity. Therefore, SMI is useful for detecting changes in the blood flow related to tumor angiogenesis, and it can be applied also in imaging techniques that allow differentiation between healthy tissue and early-stage melanoma lesions. Due to its contactless nature, low cost, compactness, low energy consumption, and self-alignment, SMI has become a novel technique that is just starting to be exploited in the field of dermatology.

Unlike other well-established techniques presented in this paper, to date, SMI is in an early stage of development regarding cancer diagnosis and prognosis; however, three experimental approaches have been proposed: Imaging, Flow Sensing, and Flow Cytometry.

### 6.1. SMI Imaging

With this scheme, it is possible to construct low-cost medical imaging systems with the inherent advantages of SMI: self-alignment and sensitivity owing to coherent detection. SMI tomography is an imaging technique that can be achieved on any diffusing media, such as the skin, by the use of modulated, frequency-shifted optical feedback in the range of few hundred kilohertz to several megahertz. Different laser sources have been employed, but quantum cascade lasers (QCL) modulated in the range of 1–5 THz have turned out to be ideal to examine structures within the superficial layer of the skin, thus allowing discrimination between healthy and pathological tissue [84]. The separation between the peaks of the time-domain SMI signal, as well as their phase and shape, are parameters related to the length of the external cavity and the complex reflectivity of the target (the skin), which allows obtaining high-resolution images from tissue grafts or phantoms. This technique has also been combined to other imaging modalities, such as confocal microscopy and MS imaging. The combination with confocal microscopy, known as Confocal Laser Feedback Tomography (CLFT) has been employed successfully with the use of Vertical Cavity Surface Emitting Lasers (VCSEL). The VCSEL aperture acted as a confocal pinhole allowing elimination of the out of focus reflections, achieving self-mixing interferograms after a raster scan. The lateral and axial resolutions are similar to the ones of confocal microscopy alone. If instead of one laser source, multiple laser sources are used at different wavelengths, SMI can be combined with MS imaging. Multi-wavelength SMI system composed of VCSEL or QCL laser sources can be used to obtain multispectral interferograms.

### 6.2. SMI Flow Sensing 

SMI-based Doppler flowmeters (SMDF), intended to monitor biological fluids in real time, have been demonstrated to be well-suited for in vivo and ex vivo applications [83,85,86,87]. This technique stands out for providing a non-invasive alternative for biomedical flow sensing integrated in a compact and inexpensive setup. It is well-known that in most skin cancer conditions, the micro-capillarity network that irrigates the skin is altered in order to supply blood to the malignant tumor in an angiogenic process. With SMI Doppler sensing, it is possible to distinguish between a highly vascularized area and normal skin. 

### 6.3. SMI Flow Cytometry

It is also worth mentioning the contribution of SMI to flow cytometry (FC) for cell type discrimination and size classification using SMI. Unlike most conventional Flow Cytometry (FC) techniques, the proposed system does not require of a fluorescence labeling pretreatment with expensive dyes that can also damage the cells samples under study [88]. Despite the existing well-established label-free FC schemes, the requirement of expensive devices, such as high-speed cameras and ultrasound transducers, and the complexity of the existing technology, are shortcomings that an SMI-based flow cytometer is able to overcome. SMI is being improved to attempt single cell characterization by measuring the SMI burst that a sole human living cell generates when it passes through the focal point of the laser source; nevertheless, some shortcomings such as the increase of size detection resolution must be overcome.

Some works on SMI that could possibly contribute to the diagnosis of skin cancer in the future are presented below.

### 6.4. Possible Contributions of SMI

Lacot et al. introduced SMI tomography in 1999 [89]; here, the image of a French 5 centime coin immersed in 1 cm of milk was obtained using a Nd3+:YAG microchip laser with a modulated frequency shift in the range of few hundred kilohertz to several megahertz.

**Figure 5:**
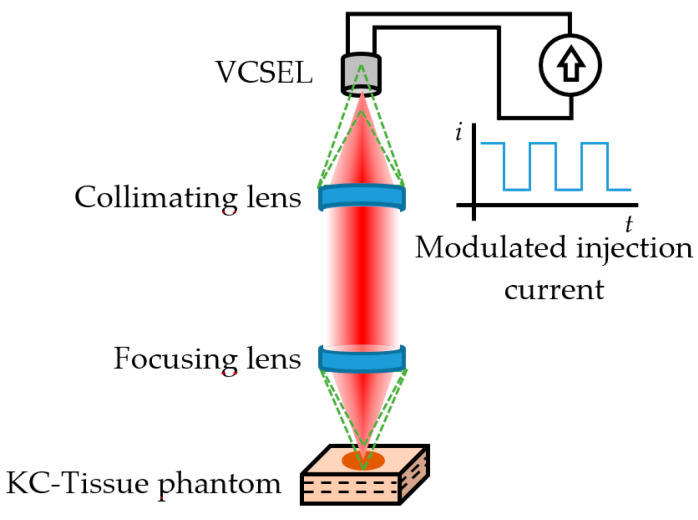
Confocal Laser Feedback Tomography as proposed by Mowla et al. [90]. The green dashed lines represent the out-of-focus reflections that are eliminated by the aperture of the 850 nm VCSEL acting as source/detector. The injection current was modulated to generate the interferometric fringes from the three-layer KC-Tissue phantom.

Later on, Mowla et al. [90] demonstrated Confocal Laser Feedback Tomography (CLFT) in a silicone-based tissue phantom with embedded inhomogeneities emulating malignant changes due to Keratinocyte Carcinoma (KC). This technique was implemented using an 850 nm VCSEL operating at 3.55 mA; the emitted beam was first collimated and later focused over the tissue phantom with a lens having a focal length of 3.1 mm and numerical aperture of 0.68, producing a laser spot diameter of 1.8 µm. The VCSEL aperture acted as a confocal pinhole allowing eliminating the out-of-focus reflections, similar to typical confocal microscopy schemes, as depicted in Figure 5.

The tissue phantom emulated three layers of healthy skin with an embedded circular region where changes in the optical properties occur due to hallmarks commonly associated to malignant KC, such as angiogenesis, abnormal cell nucleus structure, increased nuclear cell volume, increased extra cellular fluid from leaky cancerous blood vessels, and other morphological and molecular changes.

The VCSEL current was modulated at 5 kHz to generate the self-mixing interferograms. After a raster scan, the reflection level of the signal was quantified for a volume of 4.5 × 4.5 × 0.9 mm in 91 × 91 steps of 50 µm pitch (lateral resolution) and 20 µm depth (axial resolution), achieving cross-sectional images of the tissue phantom at depths of about 75 µm. Using isosurface of points at a constant signal level within the signal matrix, the boundaries of the KC were successfully represented in a 3D space. The results suggested the viability of a low-cost device for early stage skin cancer detection.

Perchoux et al. [83] developed a prototype flow sensor aimed to detect variations in the flow profile of highly vascularized skin when compared to healthy tissue. They simulate the angiogenesis process occurring due to malignant melanoma by applying a vasodilatation cream to four voluntaries. In order to observe the evolution of the skin vascularization along time, three different measurements were taken (before the application of the cream, 5 min after the application, and 25 min after the application) using an SMDF setup operating at 1300 nm. Results demonstrated that the proposed SMDF was able to distinguish the variation in the blood flow from an initial increase during the first 5 min after the application, and a subsequent drop as the effect of the cream decreases 25 min later.

**Figure 6:**
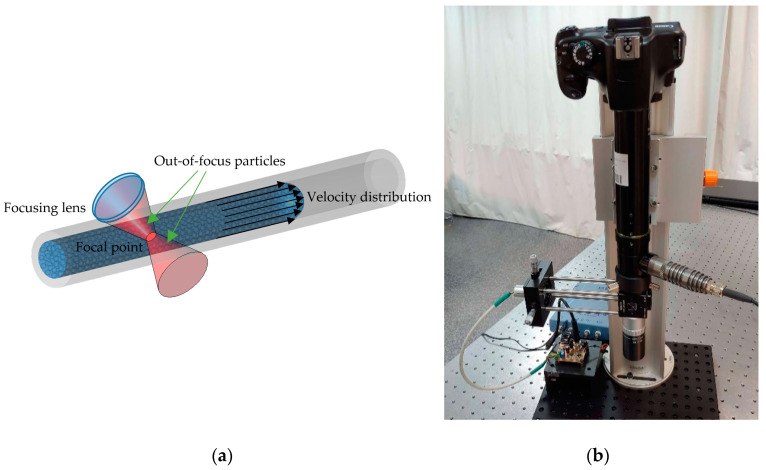
(**a**) Diagram of the interaction of a focused laser beam on particles that flow at different velocities within a capillary. (**b**) Confocal SMI-based Doppler flowmeter (SMDF) developed by Yáñez et. al. [91], which allows reconstructing the flow profile of liquids flowing in capillaries. This prototype is projected as a useful tool in the diagnosis of sprouting angiogenesis.

Yáñez et al. [91] proposed a confocal SMDF approach that allows real-time depth sectioning in turbid liquids flowing in capillaries, which is aimed at diagnosing the sprouting angiogenesis under the skin tumor. Usually, when the velocity of a turbid liquid flowing within a biological or artificial channel is evaluated by means of an SMDF, the resulting SMI signal includes the Doppler frequencies corresponding to all the velocities of the moving particles in a wide region around the focal point, as is illustrated in Figure 6a. This increases the uncertainty of the system, especially when the focal point goes deep into the first layers of the skin before reaching the abnormal vascularity. To overcome this, the confocal SMDF presented in Figure 6b restricts back-scattered photons from regions in the near and far field around the focal point to enter the laser cavity by means of a micrometric pinhole and a high numerical aperture microscope objective, thus allowing increasing the influence of ballistic photons from the focal plane on the SMI signal.

## 7. Polarimetric Imaging

The optical techniques that make use of light properties such as intensity, wavelength, and coherence, which have been previously presented, are traditionally well known. However, polarimetric imaging, a novel optical imaging technique, takes advantage of the fourth intrinsic property of light, polarization. The polarization state of the light carries information about the anisotropic properties of the tissue structure, and it can be measured simply by collecting the reflected light coming from the skin passing through a polarization state analyzer (PSA). At first, PSAs, which comprised two linear polarizers, were employed completely crossed as a qualitative improvement by dermatologists to diminish specular reflection coming from the skin surface. Later on, since the polarization of light can provide information that is not correlated to the other properties of light, it has been applied to the non-invasive analysis of the skin to characterize the morphological changes in the tissue structure. With polarimetric imaging, wide field images can be produced over a large region of the specimen, and the optical setup can be designed using inexpensive light sources such as white LEDs [92]. In addition, it can be employed with a variable wavelength illumination source to make MS polarized imaging. There are two different imaging techniques based on the measurement of polarization signature of tissues: Stokes and Mueller imaging.

Stokes imaging makes use of the complete polarization state of light to characterize skin in terms of four Stokes polarization parameters.

### 7.1. Stokes Imaging

**Figure 7:**
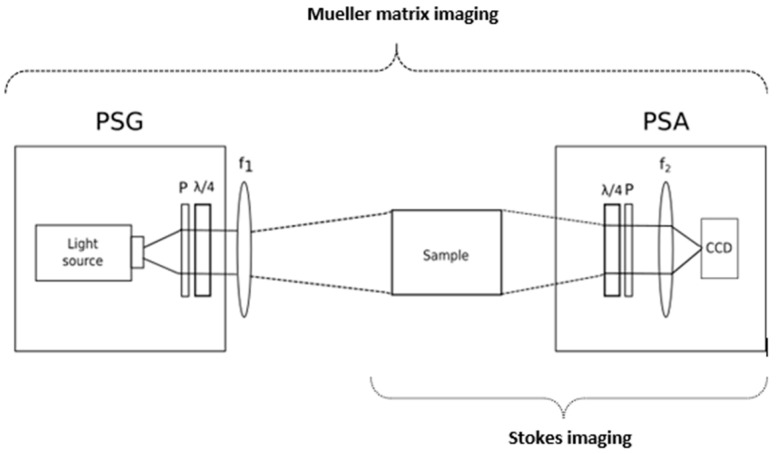
Stokes and Mueller matrix imaging (MMI) setup. The Stokes imaging setup included the sample and the polarization state analyzer (PSA). The PSA is a quarter wave plate (QWP) followed by a polarizer (P) and the optical relays (f_2_) to focus the light onto the sensor. The MMI setup consists in the previous setup with a polarization state generator (PSG). The PSG comprises a light source, a polarizer (P), and a QWP. The optical set (f1) focuses the input light on the sample.

Stokes imaging is a technique that captures the polarization state of light by measuring the four Stokes polarization parameters that fully describe it. The first Stokes parameter (S0) accounts for the intensity, the second (S1) represents the difference between horizontal and vertical polarization states, the third parameter (S2) stands for the difference between the 45° polarized and the 135° polarized light, and the fourth parameter (S3) represents the difference between right-circular (RC) polarized light and left-circular (LC) polarized light. A Stokes polarimeter is compound by a light source, a PSA, and a detector. The PSA is normally a linear polarizer followed by a quarter wave plate (QWP), as shown in Figure 7. However, in designs where speed is a requirement, such as the in vivo analysis of skin, liquid crystal variable retarders (LCVR) may substitute the QWP. By illuminating the sample with the (passive or active) light source, the PSA selects the different polarization states to be imaged: the linear 0°, 45°, 90°, and 135° together with RC and LC polarization states. From the complete polarization state, images representing other related advanced parameters can be obtained, such as the degree of polarization (DOP), the degree of linear polarization (DOLP), the degree of circular polarization (DOCP), the angle of linear polarization (AOLP), and the ellipticity. These can provide high contrast between different lesions, since the changes in melanoma/nevi structures can be correlated with the collagen contents and organization difference [93,94,95,96]. 

The Stokes algebra is suitable for all states of polarization, partially or unpolarized light, and it is compatible with the Mueller algebra. Mueller matrix imaging (MMI) consists in the measurement of the bulk tissue’s lumped polarization transfer function and provides all the possible optical polarization-dependent properties of a biological tissue such as scattering, absorption, retardance, and optical activity.

### 7.2. MMI

MMI is a technique that captures the 16-element Mueller matrix, which describes how the input Stokes vector changes upon interactions with the sample. Full determination of the matrix is obtained by using a Mueller polarimeter that comprises a Stokes polarimeter with the additional polarization state generator (PSG), which modulates the input light polarization [92]; see Figure 7. The Mueller matrix elements on their own cannot provide detailed information about a sample with unknown properties, since several polarization effects occur simultaneously. Nevertheless, there are decomposition methods that assist in the isolation of the individual properties. Several Mueller matrix decomposition methods have been proposed, although Lu-Chipman [97,98] polar decomposition may be considered as the standard. This decomposition is sequential, and it models the tissue as a sequence of depolarization, retardance, and diattenuation. The depolarization is related to the transport albedo and depends on the tissue type [99]. The retardance matrix can be decomposed into linear/circular retardation, the latter related to the concentration of biological chiral substances. The diattenuation can be extracted, but it has limited relevance in tissue analysis.

Therefore, Mueller matrix decomposition enables the discrimination between effects from multiple scattering and the retardance changes due to structural or compositional anisotropy. Having access to all this complete biophysical information through polarimetric Mueller imaging in the diagnosis of cancer has shown a huge potential and received attention toward precancer detection, where the depolarization, retardance, and diattenuation parameters extracted from the epithelium region have been used as biological metrics in different types of cancers [100,101], including skin cancer [102,103,104].

### 7.3. Contributions of Polarimetric Imaging

**Figure 8:**
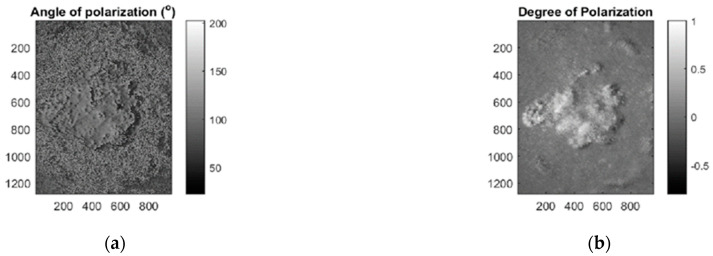
(**a**) Map of the measured AOLP (angle of linear polarization) from a melanoma lesion. (**b**) Map of DOP (degree of polarization) from the melanoma lesion. Notice the lesion region with a higher DOP than its surroundings.

Rey-Barroso et al. [93] have used advanced polarimetric parameters such as DOP (degree of polarization) and AOLP (angle of linear polarization) (Figure 8) to show the high contrast between different lesions, such as nevi and melanoma, due to the changes in the structural distribution, in specific the ones related to the change in the anisotropic media distribution such as collagen fibers. 

Ghassemi et al. [103] demonstrated that different roughness in melanoma lesions leads to abnormal polarization changes and loss of DOP, using MMI for in vivo differentiation between melanoma, benign nevi, and normal skin.

The main achievements of these works are summarized in Table 5.

## 8. Learning Algorithms for Skin Cancer Diagnosis

In parallel to the development of the aforementioned optical techniques for the improvement of skin cancer diagnosis, Computer-Aided Diagnosis (CAD) in dermatology has been empowered by the use of automatic classification algorithms [105]. In the last two decades, datasets acquired with these techniques have been employed as inputs in machine and deep learning algorithms to provide an objective judgement during the physician’s evaluation for the early detection of equivocal lesions. Although several approaches have been proposed, most of them are based on images as input data, while only a few relies on spectroscopic [106], OCT [107], or 3D [65] information. 

### 8.1. Machine Learning

Machine learning (ML) algorithms triggered the most recent advance of automated classifiers for CAD of skin lesions, especially to distinguish between melanoma, the most aggressive form, and other pigmented skin lesions. The ML process initially requires segmenting the lesion from the surrounding skin to isolate it; this might be critical, because in some cases, there is not a clear boundary limiting the lesion. Some of the segmentation algorithms used are built from Otsu’s thresholding, k-means, fuzzy c-means, and neural networks [108,109]. Most of ML approaches try to reproduce physicians’ assessments, the well-known ABCDE rule, and because of that, the next step consists in feature extraction of morphological (e.g. area, perimeter, symmetry, and eccentricity) and colorimetric (e.g., hue, saturation, lightness, and homogeneity) data from lesions [110,111,112]. Textural parameters have also shown to be very useful, since they quantify image features from a statistical approach considering image histogram, binary patterns, Gabor filters, or gray-level co-occurrence [113,114]. All these data are finally used to feed the classification models, which is the ML stage where the largest amount of resources has been addressed. 

**Figure 9:**
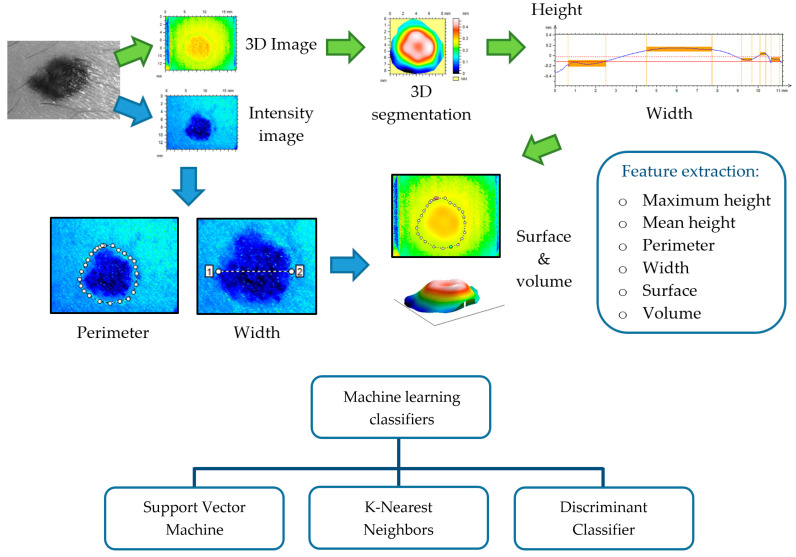
This illustrates the process of feature extraction, preceded by lesion segmentation from perilesional healthy skin, and feeding of machine learning classifiers with such features as descriptors.

There is not an ideal classification algorithm that outperforms the others as it depends on the dataset, image segmentation, and feature extraction (see Figure 9); however, in the literature, some of them have been found to provide better discrimination among skin lesions. Support Vector Machine (SVM) is the most applied classifier, as it shows the best performance [107,109,110,111,112,114,115,116,117,118] followed by k-Nearest Neighbors (KNN) [65,106,107,108,109,110,111,116,117,118], Neural Networks (NN) [110,114,115,117,119], Random Forest (RF) [107,109,113,114] and Decision Trees (DT) [65,109,110,111,117,118]. SVMs are based on statistics to build hyperplanes that maximize the distance between sets of data points [120,121]. The separators can be linear or present higher dimensionality by the use of kernel functions, leading to a wide range of SVM algorithms. The KNN classification model estimates the density distribution of the data that is determined by the distance *k* among neighbors; larger values lead to the smoothing of local features [122]. NNs are collections of highly interconnected units that interchange information that is weighted to adjust the iterative learning process [123]. DTs discriminate among data by implementing a set of rules obtained by tree derivation, i.e., starting from the initial statement (root) and ending in a class (leaf) [124]. RFs and DTs are related, since the former consists of a group of DTs that employ the classification of the different trees to set the final data discrimination [125].

ML algorithms show good performance when dealing with a few hundred features, but when features start to increase, their classification accuracy decreases. Image-based technologies are clear examples of this, since they work with millions of features due to the huge amount of pixels available in each image. In order to overcome this constraint, deep learning algorithms were developed.

### 8.2. Deep Learning

**Figure 10:**
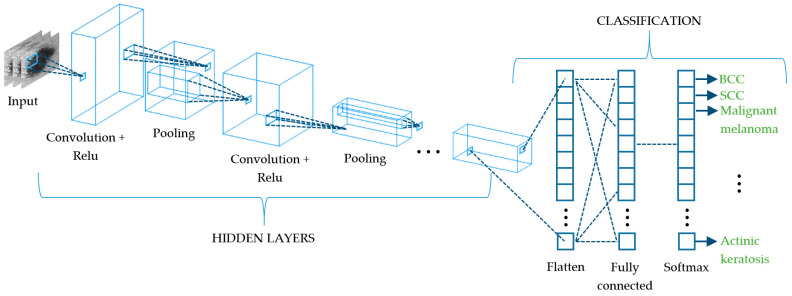
Schematic representation of a convolutional neural network (CNN) that extracts the different spatial characteristics of a skin cancer lesion at its subsequent layers.

Deep learning (DL) algorithms are considered the last important breakthrough in CAD as they are the evolution of conventional ML and state-of-the-art techniques, since most of the literature corresponds to the last two years. The vast majority of DL approaches are based on neural networks, and while some of them are applied to the whole computational diagnosis process (lesion segmentation, feature extraction, and classification) [126,127,128,129], others are used in combination with traditional ML classifiers such as SVM, KNN, or RF [130,131,132,133]. In general terms, DL consists of a network of multiple hidden layers where each of them is connected non-linearly to other hidden layers (Figure 10) and becomes accurately weighted when an optimization routine is applied [130]. This leads to very strong learning algorithms with very high accuracy levels, making it hard to choose one above the others. Some of the DL convolutional neural networks (CNN) that are being used currently are the LeNet-5 [134], AlexNet [135], VGGNet [136], GoogLeNet (Inception) [137], and ResNet [138]. LeNet-5 is a classic and straightforward CNN that comprises two sets of convolutional and average pooling layers, a flattening convolutional layer, two fully-connected layers, and a softmax classifier [134]. The AlexNet also consists of five convolutional layers, some of them followed by max-pooling layers, and three fully-connected layers with a final softmax layer [135]. The VGGNet increases the number of layers up to 19 comprising 16 convolutional layers with three fully connected layers [136]. The GoogLeNet is a deeper CNN based on the Inception architecture that considers a sequence of 22 convolutional layers, an average pooling layer together with a fully connected layer [137]. The ResNet offers an alternative to deep neural networks since they are more difficult to train. In this approach, layers are built as learning residual functions with reference to the layer inputs, producing a depth of 152 layers with lower complexity than previous deep CNN [138]. In order to take advantage of more than one CNN to discriminate among a set of skin lesions, some authors have proposed the combination of several algorithms to build what is known as ensembled CNNs [139,140,141,142]. This procedure is applied both to retrieve deep features from skin lesions and to classify them; however, they are very dependent on the amount of labeled images of the training set. Currently, this issue is faced by means of the transfer learning technique that increases the accuracy of the classification process with a limited number of training samples, being useful when the amount of them is very different from that of the test dataset [143]. In short, it improves the capability to transfer the knowledge learnt by the algorithm when dealing with a type of class to a different class.

Another approach that is being applied when dealing with limited training sets is data augmentation. Its fundamentals are to increase and balance the size of datasets between types of lesions by cropping, rotating and flipping images [127,144,145], and by applying color transformations [132,146].

## 9. Conclusions

Within this review, some of the most popular optical technologies that have been used for skin characterization and diagnosis of skin cancer are discussed. Additionally, tools based on learning algorithms aimed at helping in the classification of skin lesions are also presented. Examples of the most relevant results obtained by using each of these technologies are summarized as well as the obstacles still to be resolved and milestones to be met.

Most of the outcomes of the studies carried out with these technologies show the great potential of these optical modalities to perform non-invasive measurements of skin lesions, thus improving the current visual diagnosis of skin cancer based in the expertise of the dermatologist, namely dermoscopy. Nevertheless, histological examination following a biopsy still remains as the gold standard, as none of the former techniques outperforms this analysis, providing slightly lower sensitivity and specificity values. Some studies have suggested that the observations made through these optical modalities can be as good as the observations made by experienced dermatologists, but in general, they should not be considered a replacement for the standard diagnostic procedure, just valuable complementary tools.

Each technique has its own advantages and disadvantages. For instance, MS imaging is a very affordable technology that is suitable for in vivo and ex vivo applications, but in order to obtain information with cellular resolution, it needs to be combined with other modalities, such as confocal microscopy. Three-dimensional (3D) topography provides very precise information about volumetric characteristics and roughness, but it is very sensitive to the movement of the patient. OCT has shown great potential for skin cancer diagnosis; it provides many different modalities to explore blood flow and morphology, but there is still a trade-off among obtaining a better resolution of the images or a deeper penetration into the tissue. Confocal microscopy is the closest modality to the clinic. In fact, commercial systems that include confocal microscopes are already widely used at the patient’s bedside for the lateral margin detection of skin cancers, especially for BCC extraction surgery. There are other techniques that still may have a potential, but they have not yet been exploited for these purposes, such as SMI and polarimetric imaging. Specially, polarimetric imaging could retrieve information about structural properties that have not yet been determined, and it can be combined with MS imaging.

One possibility that has been recently suggested by authors to overcome the limited sensitivity and specificity values is the combined use of optical modalities, reaching values larger than those achieved by the sole use of one of the technologies. Furthermore, the use of such techniques in combination with tools including learning algorithms, namely machine and deep learning, can even improve a little bit more the outcomes without the need for experienced dermatologists, as they are completely automatic. Therefore, they might be very useful for non-dermatologist clinicians; e.g., to carry out screenings over large population samples.

Whether these techniques (or some of them) will succeed and replace the traditional tools for skin cancer diagnosis is still an open question that will be answered in the next coming years.

## Figures and Tables

**Table 1:** Contributions of Confocal Laser Scanning Microscopy (CLSM) to skin cancer diagnosis.

Study	Year	Skin Cancer Type	No. of Lesions	Modality	Illumination Source	No. of Images	Axial Res.	Lateral Res.	SE/SP
Guitera et al.	2009	Melanoma, Nevus	>300	RCM, commercial CLSM	Laser diode, 830 nm	>100	3–5 μm	1 μm	Mel. Light- colored: 85%/84% Pigmented: 92%/65%
Guitera et al.	2012	Melanoma, BCC, Nevus, Pigmented facial macules, Other tumors	>700	RCM, commercial CLSM	Laser diode, 830 nm	>100	3–5 μm	1 μm	100% 88.5%
Segura et al.	2009	Melanoma, BCC, SCC, Keratosis, Nevus	>150	RCM, commercial CLSM	Laser diode, 830 nm	>100	5 μm	2 μm	86.1% 95.3%
Ulrich et al.	2008	AK, perilesional healthy skin	>20	RCM, commercial CLSM	Laser diode, 830 nm	4–6 images	3–5 μm	1 μm	-
Horn et al.	2008	AK, perilesional healthy skin	30	RCM, commercial CLSM	Laser diode, 830 nm	>50	3–5 μm	1 μm	93.34% 88.34%
Gareau et al.	2008	BCC	-	FCM, mosaicing	Argon-ion laser, 488 nm	36 × 36 images for a mosaic	1.1 μm	0.25 μm	-
Gareau et al.	2009	BCC, healthy skin	>40	FCM, mosaicing	Argon-ion laser, 488 nm	45 confocal mosaics	1.1 μm	0.25 μm	96.6% 89.2%
Abeytunge et al.	2011	BCC	1	FCM, strip mosaicing	Argon-ion laser, 488 nm	31 strips for a mosaic	1.61 μm	0.33 μm	-

**Table 2:** Contributions of MS imaging to skin cancer diagnosis.

Study	Year	Skin Cancer Type	No. of Lesions	Modality	Spectral Range	No. of Images	Spectral Res.	Lateral Res.	SE/SP
Tomatis et al.	2003	Melanoma, BCC, Keratosis, Nevus, Other tumors	>500	MS, Pasive Staring imaging	400–1040 nm	17 images	40 nm	300 μm pixel size	78% 76%
Bekina et al.	2012	Papilloma, Melanoma	<10	MS, Active Staring imaging	450, 545, 660 and 940 nm	4 images	-	-	-
Jakovels et al.	2013	Bening pigmented lesions, Melanoma	>100	MS, Pasive Staring imaging	450–950 nm	51 images	10 nm	75 μm pixel size	-
Kim et al.	2016	Nevus, Acne lesion	2	MS, Smartphone-based, Active Staring imaging	440–690 nm	9 images	-	18 μm pixel size	-
Stamnes et al.	2017	Melanoma, BCC, SCC, Keratosis, Nevus	>500	MS, Active Staring imaging	365–1000 nm	10 images at different illum./acq. angles	-	25 μm pixel size	99% 93%
Delpueyo et al.	2017	Melanoma, BCC, Nevus	>400	MS, Active Staring imaging	414–995 nm	8 images at different pol. angles	-	18 μm pixel size	Melanoma: 87.2% 54.5% All: 91.3% 54.5%
Vasaturo et al.	2017	Melanoma	-	MS, Ex-vivo microscopy	420–720 nm	15 images	20 nm	1392 x 1040 pixels	-
Rey- Barroso et al.	2018	Melanoma, Nevus	>50	VIS and exNIR MS, Active Staring imaging	414–1613 nm	14 images	-	CCD: 18 μm pixel size InGaAs: 70 μm pixel size	78.6% 84.6%
Godoy et al.	2015	Melanoma, BCC, SCC, Benign pigmented lesions	>100	MS, LWIR Staring imaging	750–2500 nm	60 frames/s during 2 min	-	300 μm pixel size	95% 83%
Fioravanti et al.	2016	Primary melanoma, Metastasis, healthy skin	15	MS, Ex-vivo IR spectrometry	3330–3570 nm	None – Integrated spectral information	12 nm	-	-

**Table 3:** Contributions of 3D topography to skin cancer diagnosis.

Study	Year	Skin Cancer Type	No. of Lesions	Modality	Illumination Source	No. of Images	Axial Res.	Lateral Res.	SE/SP
Gorpas et al.	2006	Animal model tumor	1	Fringe projection	Blue laser diode 440 nm		-	4.4 μm	-
Moore et al.	2006	Multiple cancers	-	Fringe projection	He-Ne laser 633 nm	-	-	-	-
Ares et al.	2014	-	-	Fringe projection	He-Ne laser 633 nm	-	-	-	-
Rey-Barroso et al.	2014	Melanoma, BCC, Nevus	>170	Fringe projection	He-Ne laser 633 nm	-	-	-	80.0% 76.7%
Korn et al.	2017	None – body parts exposed to sun	5 body sites in 20 patients	3D optical profilometry	Hallogen lamp	8 images	0.005 μm	1.1 μm	-

**Table 4:** Contributions of Optical Coherence Tomography (OCT) to skin cancer diagnosis.

Study	Year	Skin Cancer Type	No. of Lesions	Modality	Laser Source	No. of Images	Axial Res.	Lateral Res.	SE/SP
Korde et al.	2008	AK, body parts exposed to sun	4 body sites in 112 patients	FD-OCT	1310 nm	1344 OCT images	12 μm	12 μm	86% 83%
Themstrup et al.	2016	None–healthy skin subject to different conditions	-	OCTA, commercial OCT	1305 nm	-	5 μm	7.5 μm	-
Weissman et al.	2004	None–healthy skin to compare with BCC	-	FD-OCT, commercial OCT	LEDs, 1300 nm	47 OCT images	5 μm	3 μm	-

**Table 5:** Contributions of polarimetric imaging to skin cancer diagnosis.

Study	Year	Skin Cancer Type	No. of Lesions	Modality	Illuminationsource	No. of Images	Polarization Configurations	Lateral Res.	SE/SP
Rey- Barroso et al.	2019	MM, Nevus	40	Polarized MS imaging	LEDs, 414–995 nm	8 images	0°, 45° and 90°	18 μm pixel size	-
Ghassemi et al.	2012	MM, Nevus, healthy skin	>20	Stokes imaging	Tricolor LED-based, Hemisferical	16 images at illum. angles 0°, 24°, and 49°	0°, 45°, 90° and 135°	-	-

## Data Availability

No new data were created or analyzed in this study. Data sharing is not applicable to this article.
